# Online Impact and Presence of a Specialized Social Media Team for the Journal of Neurosurgery: Descriptive Analysis

**DOI:** 10.2196/17741

**Published:** 2020-05-19

**Authors:** Joseph R Linzey, Faith Robertson, Ali S Haider, Christopher Salvatore Graffeo, Justin Z Wang, Gillian Shasby, Naif M Alotaibi, Aaron A Cohen-Gadol, James T Rutka

**Affiliations:** 1 Department of Neurosurgery University of Michigan Ann Arbor, MI United States; 2 Department of Neurosurgery Massachusetts General Hospital and Harvard Medical School Boston, MA United States; 3 Department of Industrial and Systems Engineering Texas A&M University College Station, TX United States; 4 Department of Neurologic Surgery Mayo Clinic Rochester, MN United States; 5 Division of Neurosurgery Faculty of Kinesiology and Physical Education University of Toronto Toronto, ON Canada; 6 Journal of Neurosurgery Publishing Group Charlottesville, VA United States; 7 Department of Neurological Surgery Indiana University Indianapolis, IN United States

**Keywords:** social media, Twitter, Facebook, research dissemination

## Abstract

**Background:**

Social media use continues to gain momentum in academic neurosurgery. To increase journal impact and broaden engagement, many scholarly publications have turned to social media to disseminate research. The Journal of Neurosurgery Publishing Group (JNSPG) established a dedicated, specialized social media team (SMT) in November 2016 to provide targeted improvement in digital outreach.

**Objective:**

The goal of this study was to examine the impact of the JNSPG SMT as measured by increased engagement.

**Methods:**

We analyzed various metrics, including impressions, engagements, retweets, likes, profile clicks, and URL clicks, from consecutive social media posts from the JNSPG’s Twitter and Facebook platforms between February 1, 2015 and February 28, 2019. Standard descriptive statistics were utilized.

**Results:**

Between February 2015 and October 2016, when a specialized SMT was created, 170 tweets (8.1 tweets/month) were posted compared to 3220 tweets (115.0 tweets/month) between November 2016 and February 2019. All metrics significantly increased, including the impressions per tweet (mean 1646.3, SD 934.9 vs mean 4605.6, SD 65,546.5; *P*=.01), engagements per tweet (mean 35.2, SD 40.6 vs mean 198.2, SD 1037.2; *P*<.001), retweets (mean 2.5, SD 2.8 vs mean 10.5, SD 15.3; *P*<.001), likes (mean 2.5, SD 4.0 vs mean 18.0, SD 37.9; *P*<.001), profile clicks (mean 1.5, SD 2.0 vs mean 5.2, SD 43.3; *P*<.001), and URL clicks (mean 13.1, SD 14.9 vs mean 38.3, SD 67.9; *P*<.001). Tweets that were posted on the weekend compared to weekdays had significantly more retweets (mean 9.2, SD 9.8 vs mean 13.4, SD 25.6; *P*<.001), likes (mean 15.3, SD 17.9 vs mean 23.7, SD 70.4; *P*=.001), and URL clicks (mean 33.4, SD 40.5 vs mean 49.5, SD 117.3; *P*<.001). Between November 2015 and October 2016, 49 Facebook posts (2.3 posts/month) were sent compared to 2282 posts (81.5 posts/month) sent between November 2016 and February 2019. All Facebook metrics significantly increased, including impressions (mean 5475.9, SD 5483.0 vs mean 8506.1, SD 13,113.9; *P*<.001), engagements (mean 119.3, SD 194.8 vs mean 283.8, SD 733.8; *P*<.001), and reach (mean 2266.6, SD 2388.3 vs mean 5344.1, SD 8399.2; *P*<.001). Weekend Facebook posts had significantly more impressions per post (mean 7967.9, SD 9901.0 vs mean 9737.8, SD 19,013.4; *P*=.03) and a higher total reach (mean 4975.8, SD 6309.8 vs mean 6108.2, SD 12,219.7; *P*=.03) than weekday posts.

**Conclusions:**

Social media has been established as a crucial tool for the propagation of neurosurgical research and education. Implementation of the JNSPG specialized SMT had a demonstrable impact on increasing the online visibility of social media content.

## Introduction

Social media use continues to gain momentum in both the general public and in academic medicine. When the Pew Research Center began tracking social media statistics in 2005, 5% of Americans were technology adopters, whereas 72% of the public currently uses some variation of social media (eg, Facebook, Twitter, Youtube, LinkedIn, Instagram) [1]. These digital applications are multidimensional tools that—in addition to aiding personal relationships and providing entertainment—can be leveraged to disseminate research to both medical professionals and patient populations, strengthen professional networks, and promote increased connectivity within a professional field. The use of social media to improve literature dissemination, foster a digital presence, and enhance programs’ rankings has been especially prominent for surgical specialties, with many recent publications and digital topic trends in general [2,3], cardiothoracic [4], urology [5], and plastic surgery [6], as well as other subspecialties.

For neurosurgery specifically, there is particular interest in engaging younger surgeons, publicizing literature, and enhancing more global collaborations. The 2018 article “Millennials in neurosurgery: Is there hope?” [7] expressed concern for the traits and opinions of this new generation, but millennials were commended for their increased use and familiarity with communications, media, and technology along with an expectation to use these tools daily to make their work more efficient. Importantly, as social media tools are embraced by all demographics, and the highest adopters are young professionals (88% of 18- to 29-year-olds), social media provides an opportunity to further engage and train the new generation. Furthermore, we are experiencing a shift in the paradigm for evaluating the impact of research. Academic science and biomedicine publications have been historically ranked by citation-based metrics (eg, H-index, impact score); however, altmetrics are gaining prevalence within medicine [8,9]. Altmetrics are an alternative set of quantitative and qualitative scores such as attention, mentions, and retweets that assess public engagement. In 2017, Wang et al [10] conducted a qualitative analysis of the highest trending works in neurosurgery along with a correlation analysis with their social media metrics. In considering altmetric scores, they found an average score of 4.7 (SD 22.4); journals with a social media account had significantly higher altmetric scores for their articles than journals without such an account (*P*<.001). The top 100 neurosurgical articles in altmetrics belonged primarily to those with active accounts, with *Journal of Neurosurgery* ranking the highest (33%), followed by *Neurosurgery* (29%) [10]. ResearchGate is another digital platform that is widely used to share articles, increase neurosurgical networking, and facilitate research collaboration [9]. These digital partnerships are continuing to grow and influence how we conduct research.

Overall, social media presence for academic journals is important for greater outreach and engagement. The *Journal of Neurosurgery* Publishing Group (JNSPG) established a dedicated social media team (SMT) to provide targeted improvement of digital outreach. The goal of the present study was to examine the impact of establishing a dedicated SMT and elucidate the characteristics of Twitter and Facebook posts that optimize audience engagement. By providing a clearer understanding of how post timing, images, hashtags, and more influence interaction, these data can inform educational and business strategies for social media influencers and other academic journals.

## Methods

### The Social Media Team

The JNSPG SMT was created on November 1, 2016 and is comprised of editors who examine both new publications and previously published articles in the core and subtopic journals of the JNSPG. There have been 4-6 social media editors, all of whom are either neurosurgical residents or senior medical students committed to a career in neurosurgery. They identified interesting articles and appealing or representative figures and images, and created a brief headline or summary of the publication with hashtags corresponding to the article type. Each editor was responsible for creating content for 1-3 hashtags for the upcoming week. Post content was checked and verified by a social media manager who was also a neurosurgical resident. The posts were scheduled to be published via Hootsuite software (Hootsuite Inc, Vancouver, BC, Canada) [11] at various times throughout the day. Editors were also responsible for generating visual abstracts on behalf of the journal and microblogging using their personal accounts to comment on occasional articles. Visual abstracts underwent an extra layer of scrutiny before publication. The journal’s editorial and staff teams analyzed the visual abstracts to ensure congruency with the manuscript’s original content and appeal of the visual components of the abstract.

### Study Design

We analyzed metrics from consecutive social media posts from the JNSPG’s Twitter and Facebook platforms between February 1, 2015 and February 28, 2019. All data were obtained from the analytics collected by Twitter and Facebook. Since all data used were publicly accessible, Institutional Review Board approval was not needed. Additionally, since no patient information was accessed, no patient consent was sought.

### Social Media Data

#### Twitter

The variables collected describing the Twitter posts included the content of the tweet, the date and time the tweet was posted, and the number of impressions (number of times a post was viewed by a user, whether the post was clicked on or not), engagements (number of times a post was clicked on to magnify the image or text or view a video), retweets, likes, profile clicks, and URL clicks each post received. The definitions for each metric were obtained from the Twitter analytics data we received during data abstraction.

**Figure 1:**
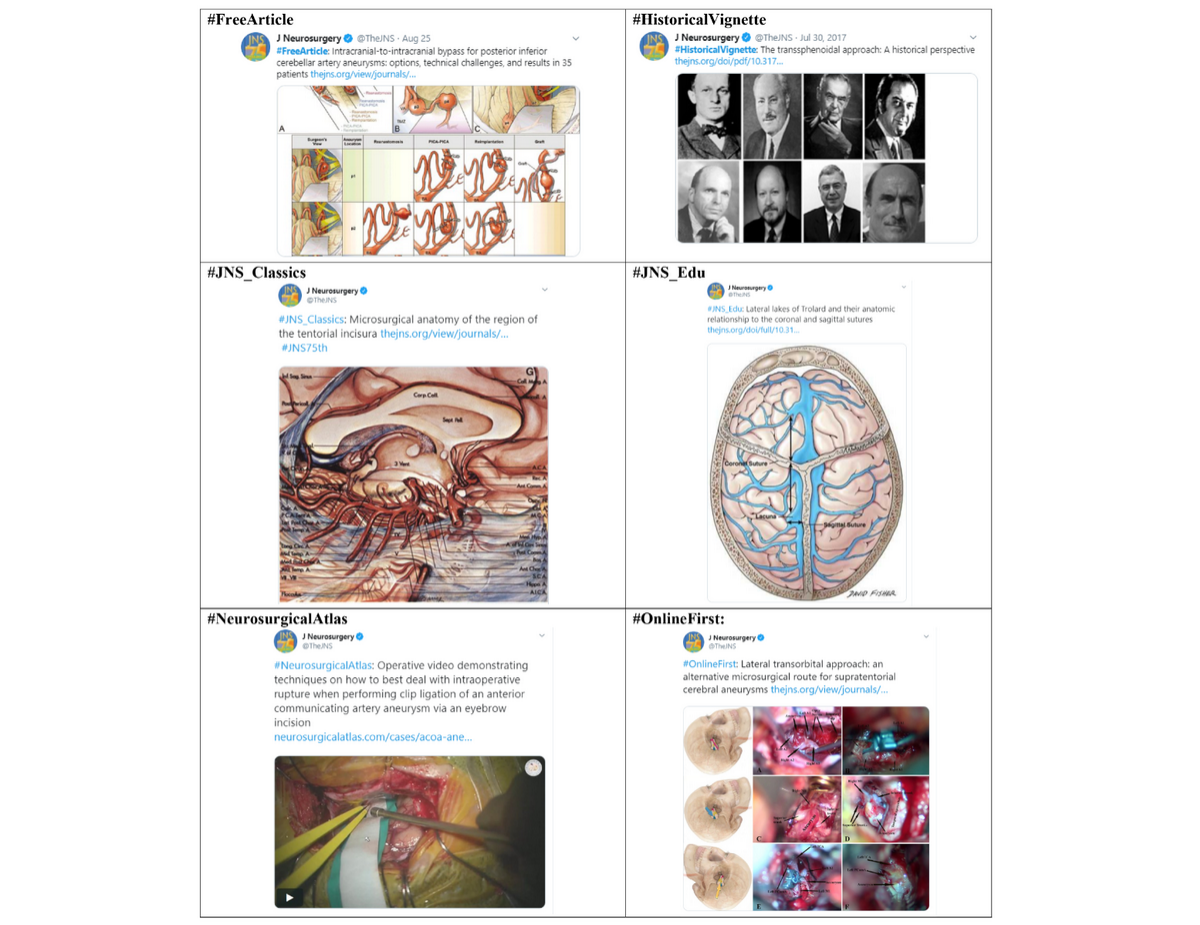
Examples of different types of Journal of Neurosurgery Publishing Group (JNSPG) Twitter posts.

The text of each tweet was searched for hashtags and divided into 14 different categories: #FreeArticle, #HistoricalVignette, #JNS_Classics, #JNS_Edu, #NeurosurgicalAtlas, #OnlineFirst, #OperativeVideo, #VideoAbstract, #VisualAbstract, #JNS_History, #GoogleAlerts, #JNS75th, #NeurosurgicalFocus, and other (see Figure 1 and Multimedia Appendix 1). The date the content was posted was dichotomized into weekdays (Monday through Friday) and weekends (Saturday and Sunday). The time the tweet was posted was also split into content sent during the workday (7 am to 5 pm) and after the workday (5:01 pm to 6:59 am) in Eastern Standard Time/Eastern Daylight Savings Time.

#### Facebook

The variables collected describing the Facebook posts included the content of the post, date and time the content was posted, number of impressions (number of times a post was viewed by a user, whether the post was clicked on or not), number of engagements (number of times a post was clicked on to magnify the image or text or view a video), and total number of unique people who received each post. The definitions for each metric were obtained from the Facebook analytics data we received during data abstraction

The text of each Facebook post was searched for hashtags and divided into 13 different categories: #FreeArticle, #HistoricalVignette, #JNS_Classics, #JNS_Edu, #NeurosurgicalAtlas, #OnlineFirst, #OperativeVideo, #VideoAbstract, #VisualAbstract, #JNS_History, #JNS75th, #NeurosurgicalFocus, and Other. The date the content was posted was dichotomized into weekdays (Monday through Friday) and weekends (Saturday and Sunday). The time the Facebook post was sent was also split into content sent during the workday (7 am to 5 pm) and after the workday (5:01 pm to 6:59 am) in Eastern Standard Time/Eastern Daylight Savings Time.

### Statistical Analysis

Twitter and Facebook posts were dichotomized into pre- and postintervention groups using the date that the social media team was initiated (November 1, 2016) as the intervention start time. Standard univariate descriptive statistical analyses, including *t* tests and one-way analysis of variance, were performed, and the data are summarized as means and SD. *P* values are based on two-sided tests, and values less than .05 were considered significant. All data were analyzed using SAS 9.4 software (SAS Institute Inc, Cary, NC, USA).

## Results

### Twitter

Between February 2015 and February 2019, a total of 3390 tweets were posted from the JNSPG Twitter account. In the preintervention period (February 2015 to October 2016), 170 tweets or 8.1 tweets per month were posted. After implementation of the SMT, 3220 tweets or 115.0 tweets per month were posted between November 2016 and February 2019.

The impressions per tweet significantly increased following creation of the SMT (Table 1), and interactions with Twitter content also increased significantly, including average engagements, retweets, and likes per tweet. Users were also significantly more likely to click on the JNSPG’s Twitter profile after SMT implementation. Importantly, readers were significantly more likely to click on the URLs included in the tweet, which took the user to the original journal article.

**Table 1:** Twitter analytics before and after creation of a social media team.

Metric	Before social media team, mean (SD)	After social media team, mean (SD)	*P* value
Impressions per tweet	1646.3 (934.9)	4605.6 (65546.5)	.01
Engagements per tweet	35.2 (40.6)	198.2 (1037.2)	<.001
Retweets per tweet	2.5 (2.8)	10.5 (15.3)	<.001
Likes per tweet	2.5 (4.0)	18.0 (37.9)	<.001
Profile clicks per tweet	1.5 (2.0)	5.2 (43.3)	<.001
URL clicks per tweet	13.1 (14.9)	38.3 (67.9)	<.001

**Figure 2:**
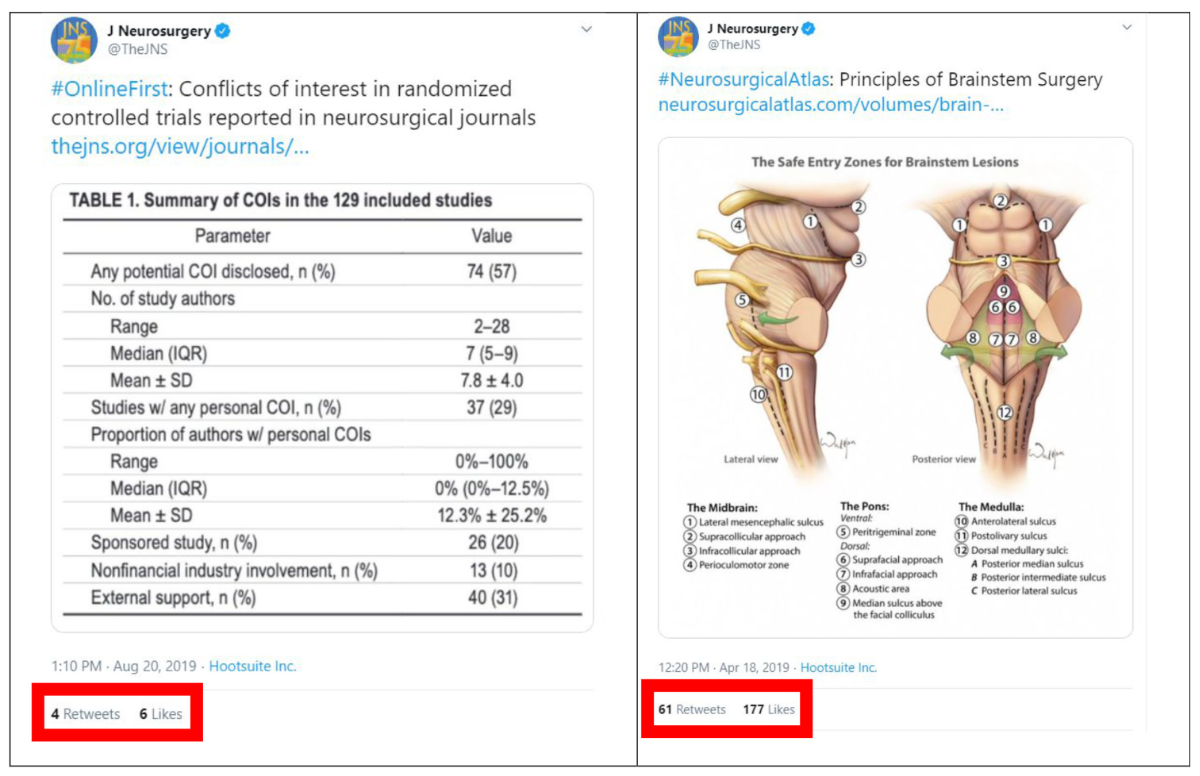
Differences in retweets and likes between #OnlineFirst and #NeurosurgicalAtlas.

Interactions between JNSPG tweets varied significantly depending on the content and hashtags used for different tweets. In almost all categories (engagements, retweets, likes, profile clicks, and URL clicks per tweet), tweets with the hashtags #JNS_Edu, #NeurosurgicalAtlas, #OperativeVideo, and #VisualAbstract were significantly more likely to receive high levels of interaction from users (*P*<.001, Table 2). Content in the #OnlineFirst, #GoogleAlert, #VideoAbstract, or Other categories was less likely to elicit similarly high levels of interactions (Figure 2).

**Table 2:** Twitter analytics, mean (SD) per tweet, by hashtag.

Hashtag	N	Impressions	Engagements	Retweets	Likes	Profile clicks	URL clicks
#FreeArticle	701	3750.6 (2892.6)	215.6 (231.2)	11.7 (9.1)	19.6 (16.7)	4.5 (5.9)	43.8 (37.9)
#Historical Vignette	60	5157.8 (4489.8)	234.5 (193.9)	14.7 (10.7)	24.5 (16.2)	5.0 (6.0)	30.0 (21.2)
#JNS_Classics	66	3518.0 (3123.7)	217.4 (385.8)	12.1 (12.0)	22.0 (25.7)	5.1 (9.5)	27.7 (29.5)
#JNS_Edu	15	6159.1 (2236.1)	503.7 (326.3)	24.1 (7.3)	39.7 (12.8)	9.9 (7.2)	65.7 (46.9)
#NeurosurgicalAtlas	220	6726.9 (4147.6)	447.7 (385.5)	23.6 (13.8)	48.4 (27.9)	11.5 (13.1)	83.6 (68.0)
#OnlineFirst	1173	3174.2 (2824.2)	169.3 (214.1)	9.5 (8.3)	15.1 (14.3)	3.9 (5.7)	32.5 (36.5)
#OperativeVideo	89	4832.3 (22936.4)	724.4 (6080.7)	14.3 (68.9)	34.2 (199.2)	29.8 (257.3)	55.6 (318.5)
#VideoAbstract	111	1863.1 (1105.7)	35.5 (31.1)	3.5 (1.9)	5.0 (3.6)	1.5 (1.8)	10.0 (8.2)
#Visual Abstract	12	8049.3 (3555.6)	347.8 (173.5)	25.8 (13.3)	32.3 (16.8)	10.1 (4.4)	43.5 (28.9)
Other	588	8873.8 (153016.6)	103.1 (171.5)	6.8 (9.9)	9.0 (13.2)	3.7 (6.9)	27.3 (39.7)
#JNSHistory	15	3611.3 (2406.4)	161.7 (146.0)	10.1 (7.3)	16.5 (15.2)	5.7 (4.9)	7.3 (10.5)
#GoogleAlerts	405	1620.8 (1848.0)	47.2 (70.3)	4.0 (5.5)	6.2 (8.4)	1.6 (3.7)	27.2 (41.1)
#JNS75th	15	5277.2 (2087.4)	255.2 (141.2)	16.0 (10.9)	32.1 (18.7)	10.7 (6.4)	23.3 (21.7)
#NeurosurgicalFocus	20	4435.7 (1860.7)	253.1 (194.3)	13.1 (9.2)	28.9 (18.7)	7.4 (5.2)	39.8 (28.6)
*P* value	.98	<.001	<.001	<.001	<.001	<.001	<.001

Users were significantly more likely to retweet content that was posted from the JNSPG Twitter account after work hours (Table 3) compared to content posted during work hours. However, the average number of impressions, engagements, likes, profile clicks, or URL clicks did not significantly vary based on the time of day the tweet was posted. Tweets that were posted on the weekend compared to weekdays had significantly more retweets, likes, and URL clicks per tweet (Table 3). However, there was no significant difference in the average number of impressions, engagements, or profile clicks based on whether a tweet was posted on a weekday or during the weekend.

**Table 3:** Twitter analytics, mean (SD), as a function of time and day.

Analytic	Tweet sent during work hours (n=2024)	Tweet sent after work hours (n=1366)	*P* value	Weekday tweets (n=2626)	Weekend tweets (n=764)	*P* value
Impressions per tweet	3186.3 (3107.6)	6340.3 (10,0562.0)	.25	4517.8 (72,448.4)	4248.8 (8368.4)	.85
Engagements per tweet	165.6 (234.0)	226.3 (1567.5)	.16	157.8 (232.0)	301.1 (2084.2)	.06
Retweets per tweet	9.3 (9.7)	11.4 (20.4)	<.001	9.2 (9.8)	13.4 (25.6)	<.001
Likes per tweet	16.0 (18.1)	19.0 (54.1)	.05	15.3 (17.9)	23.7 (70.4)	.001
Profile clicks per tweet	3.9 (6.3)	6.6 (66.1)	.14	4.0 (7.0)	8.5 (87.9)	.16
URL clicks per tweet	35.5 (42.5)	39.3 (91.0)	.15	33.4 (40.5)	49.5 (117.3)	<.001

### Facebook

Between November 2015 and February 2019, a total of 2331 Facebook posts were sent from the JNSPG Facebook account. Between November 2015 and October 2016, when the specialized SMT was created, 49 posts or 2.3 posts per month were sent. Between November 2016 and February 2019, after implementation of the SMT, 2282 posts or 81.5 posts per month were sent.

After implementation of the dedicated SMT, average impressions for Facebook posts, average engagements, and average reach per post increased significantly (Table 4).

Mirroring the trend found in the Twitter data, users were most likely to engage with posts with the hashtags #JNS_Edu, #NeurosurgicalAtlas, and #OperativeVideo compared to hashtags such as #OnlineFirst, #VideoAbstract, and #JNS_History (*P*<.001, Table 5). These same post types had significantly more impressions and a greater reach among Facebook users (Figure 2).

There were no significant differences in the number of impressions, engagements, or total reach of Facebook posts based on whether the post was sent during work hours or after work hours (Table 6). However, Facebook posts sent on the weekend compared to weekdays were significantly more likely to have a higher average number of impressions per post and higher total reach. The average number of engagements per post did not vary between weekend and weekday posts (Table 6).

**Table 4:** Facebook analytics, mean (SD), before and after creation of a social media team.

Analytic	Before social media team (n=49)	After social media team (n=2282)	*P* value
Impressions per post	5475.9 (5483.0)	8506.1 (13,113.9)	<.001
Engagements per post	119.3 (194.8)	283.8 (733.8)	<.001
Total reach per post	2266.6 (2388.3)	5344.1 (8399.2)	<.001

**Table 5:** Facebook post analytics, mean (SD), by hashtag.

Hashtag	N	Impressions	Engagements	Total reach
#FreeArticle	670	9088.3 (12012.8)	297.8 (501.6)	5644.3 (7379.7)
#Historical Vignette	57	7769.6 (6208.1)	203.1 (195.1)	229.1 (389.7)
#JNS_Classics	62	5644.3 (7379.7)	4862.3 (3600.6)	4849.3 (4894.9)
#JNS_Edu	17	21131.5 (12633.4)	735.7 (529.5)	12474.6 (7441.6)
#NeurosurgicalAtlas	220	11997.4 (8087.2)	442.0 (356.6)	7718.1 (4896.9)
#OnlineFirst	960	6677.1 (6365.1)	196.1 (259.5)	4197.7 (3925.4)
#OperativeVideo	83	13890.4 (40761.4)	653.4 (2949.7)	8787.3 (26849.9)
#VideoAbstract	8	4468.6 (2696.0)	141.4 (109.6)	2863.9 (1829.5)
#Visual Abstract	7	8796.0 (7721.9)	228.0 (296.5)	5402.0 (4650.2)
Other	208	8360.3 (20484.6)	303.3 (1005.6)	4964.1 (13524.1)
#JNSHistory	11	4046.7 (2941.9)	128.8 (95.3)	2629.2 (1957.6)
#JNS75th	8	8146.1 (4647.8)	294.6 (241.4)	5462.6 (3079.1)
#NeurosurgicalFocus	20	8428.2 (6108)	319.5 (329.0)	5592.9 (3881.2)
*P* value	N/A^a^	<.001	<.001	<.001

^a^N/A: not applicable.

**Table 6:** Facebook post analytics, mean (SD), as a function of time and day.

Analytic	Posts sent during work hours (n=1700)	Posts sent after work hours (n=631)	*P* value	Weekday posts (n=1706)	Weekend posts (n=625)	*P* value
Impressions per post	8533.0 (12,758.6)	8198.3 (13,659.0)	.59	7967.9 (9901.0)	9737.8 (19,013.4)	.03
Engagements per post	284.3 (749.5)	269.8 (663.1)	.65	259.2 (456.0)	338.1 (1183.6)	.10
Total reach per post	5330.5 (8107.4)	5142.0 (8904.7)	.64	4975.8 (6309.8)	6108.2 (12,219.7)	.03

## Discussion

### Principal Findings

Social media, especially Twitter and Facebook, are increasingly utilized for the dissemination of neurosurgical research. There are many potential strategies for journals to increase their social media presence, including frequent postings, the creation of a general marketing team, and the creation of a specialized SMT. Our study illustrates the success of implementing a specialized SMT for the JNSPG. All key outcomes (the quantity of content published, impressions, likes, retweets, engagements, profile clicks, URL clicks, and total reach) were significantly increased after development of a dedicated, specialized SMT.

Social media use among neurosurgeons, neurosurgical departments, and neurosurgical journals has increased exponentially over the past few years [12-17]. With the rise in social media use among neurosurgeons, there is an increased need for neurosurgical journals to produce regular, high-quality content. Altmetric [18] has been established as a grading mechanism for determining article impact on social media platforms [19]. It has been noted that articles published in journals with a robust social media presence had higher altmetric scores, further demonstrating the meaningful role of a specialized SMT in propagating an article’s online impact [10]. In addition, multiple studies have reported a correlation between an active social media presence and increased research productivity among academic medical institutions [12,13,20,21]. This provides evidence that traditional bibliometrics also benefit when an article has an impact on social media, with the presumption that the article is read, downloaded, and engaged with more due to its greater online reach.

Given the complex and unique knowledge base from which neurosurgery draws, it would be difficult for a general marketing team to develop the expertise necessary to produce content that engages and challenges the neurosurgical community. There is a distinct need for a specialized group of neurosurgical students, trainees, and attending surgeons to help create and moderate the content on the social media platforms of neurosurgical journals. Given the large amount of time needed to manage a major social media presence on top of a busy clinical practice, it is necessary to maintain a fairly large team of volunteers to appropriately divide the workload. For many trainees, the ability to “get their foot in the door” and work directly with a specialized journal in their field is sufficient motivation to provide the enthusiasm needed for sustained teams with necessary longevity. After the JNSPG created their SMT, there was a significant increase in all metrics that indicate greater engagement with the posts.

There was a significant difference in the engagement level based on the different types of posts and hashtags used. Universally, posts with impressive illustrations or videos (#JNS_Edu, #NeurosurgicalAtlas, #OperativeVideo) were significantly more likely to produce high engagement levels compared to other, less visually appealing posts (#OnlineFirst, #GoogleAlert, or Other). Having a specialized, dedicated team that is focused on optimizing the visual presentation of social media content and knowing what specific types of content are most likely to resonate with the neurosurgical community is imperative. Additionally, if feasible, it may benefit journals and institutions to have staff dedicated to medical illustrations.

Overall, there was no significant difference in the average number of impressions, likes, profile clicks, URL clicks, or total reach for Twitter or Facebook content based on whether the post was sent during typical work hours or after hours. There were significantly more retweets for after-hour posts. However, there were significantly more retweets, likes, and URL clicks for Twitter posts, along with more impressions and a higher total reach for Facebook posts if they were posted on the weekend compared to a weekday. While the time of day may not affect the likelihood of a surgeon perusing social media, these results indicate that neurosurgeons are able to take more time to not only quickly like or retweet interesting content on the weekend, but to also spend additional time engaging with the original research articles by clicking on the URLs embedded in the tweets. The time of day that content is posted may not significantly affect neurosurgeons’ engagement patterns because their busy operative schedules may allow only sporadic scrolling through their feeds at random times of the day. However, on weekends, many surgeons have extra time to more fully engage with interesting manuscripts and images that are produced for social media. Again, a dedicated SMT can take advantage of this knowledge to target especially high-impact content to the weekend when a neurosurgeon is most likely to have sufficient time to engage with the social media content.

As online resources and social media continue to play a greater role in research dissemination and clinical trainee education, it is important to emphasize the need for free educational sources that can be easily distributed via social media channels. Online educational resources such as The Neurosurgical Atlas have partnered with neurosurgical journals, including *Journal of Neurosurgery* and *Operative Neurosurgery*, to create an interactive and comprehensive learning tool for distribution on social media [22]. This educational collaboration has provided access to large amounts of instructive material to learners anywhere in the world without any direct costs. Specialized teams of social media editors can sift through and collate the vast amounts of educational data for optimal distribution.

As the *Journal of Neurosurgery* SMT continues to grow and develop, further research initiatives will be pursued. The benefits of visual abstracts, specifically in manuscripts without compelling visual figures, will be examined. Further research is needed to determine the benefit of operative videos compared to static operative or illustrated images. Finally, additional work will be pursued to delineate the relationship between the level of engagement and the time of day or day of the week when content is posted taking the worldwide viewership into account.

### Limitations

The main limitation of this study is that the data are from a single, neurosurgical journal’s experience. In the future, additional studies that compare and contrast the experiences of multiple journals with and without SMTs would strengthen the results presented in this study. Additionally, this study analyzed the JNSPG’s experience with only Twitter and Facebook. Many other journals and neurosurgical departments utilize Instagram and other social media platforms that are not reflected in these analyses. Furthermore, there is currently no strong, accepted method to correlate increased social media visibility with traditional bibliometric data. Altmetrics utilize social media engagement levels to create a score, but it is unclear if higher altmetric scores correlate with more citations in neurosurgical research. We found an association between post timing and post engagement; however, we do not have any data on when the posts were actually viewed by the user. This association is further hindered by the large international following of the JNSPG’s social media platforms, which introduces variability with different time zones. Additionally, we do not have any data on the specific metrics resulting from individual members of the SMT using their personal accounts to comment or boost the visibility of specific posts. Given the relative lack of followers that individual members of the SMT have compared to the *Journal of Neurosurgery* account, we are relatively confident that these microblogs did not significantly skew the data.

### Conclusions

Social media is established as a crucial tool for the propagation of neurosurgical research and education. Implementation of a specialized SMT has a demonstrable impact on increasing the online visibility of social media content.

## Appendix

Multimedia Appendix 1 Additional examples of the 14 categories of Journal of Neurosurgery Publishing Group (JNSPG) Twitter posts.

## Abbreviations

JNSPG: Journal of Neurosurgery Publishing Group
SMT: social media team
