# Supplementary material for: Online Impact and Presence of a Specialized Social Media Team for the Journal of Neurosurgery: Descriptive Analysis
Source: J Med Internet Res. 2020 May 19;22(5):e17741. doi: 10.2196/17741 (PMC7267990; doi:10.2196/17741)

#OperativeVideo

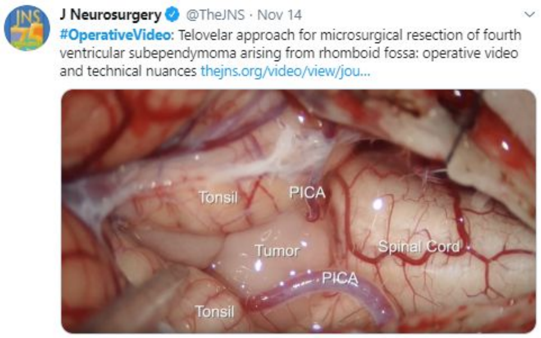

#VideoAbstract

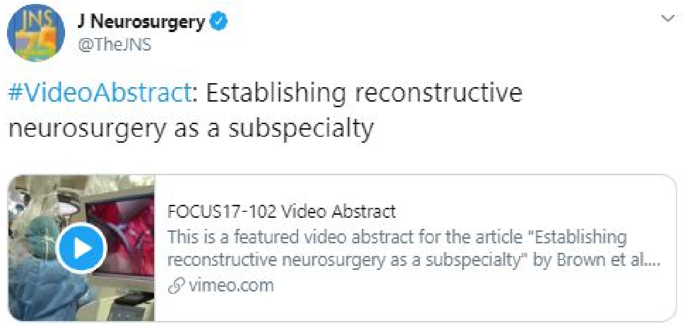

#VisualAbstract

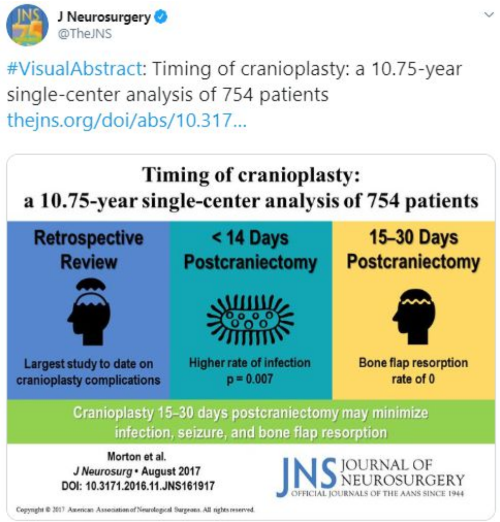

#JNS_History

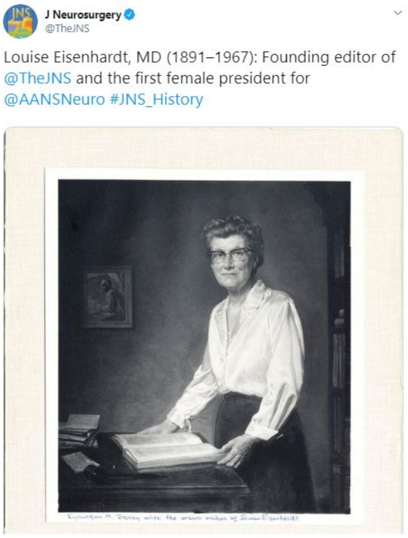

#GoogleAlerts

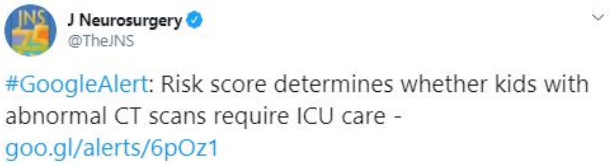

#JNS75th

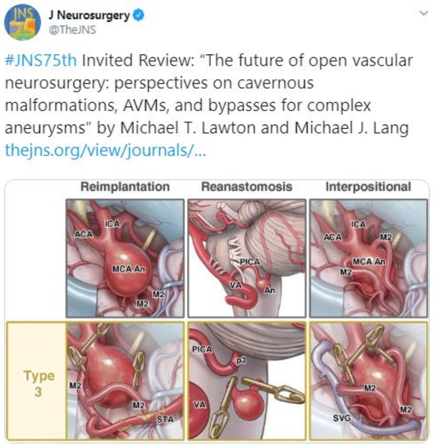

#NeurosurgicalFocus

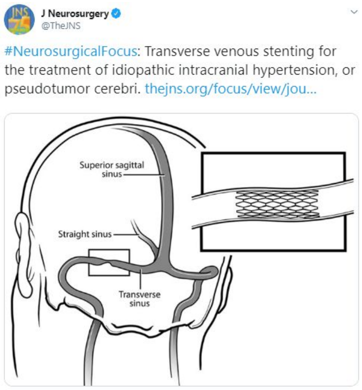

Supplement: Multimedia Appendix 1 [file jmir_v22i5e17741_app1.pdf]
